# Quantitative proteomic analysis of HER2 protein expression in PDAC tumors

**DOI:** 10.1186/s12014-024-09476-7

**Published:** 2024-03-20

**Authors:** Jamie Randall, Allison L. Hunt, Aratara Nutcharoen, Laura Johnston, Safae Chouraichi, Hongkun Wang, Arthur Winer, Raymond Wadlow, Jasmine Huynh, Justin Davis, Brian Corgiat, Nicholas W. Bateman, John F. Deeken, Emanuel F. Petricoin, Thomas P. Conrads, Timothy L. Cannon

**Affiliations:** 1 Inova Schar Cancer Institute, Inova Health System, 8081 Innovation Park Dr, Fairfax, VA 22031 USA; 2 Women’s Health Integrated Research Center, Women’s Service Line, Inova Health System, 3289 Woodburn Rd, Annandale, VA 22042 USA; 3 Gynecologic Cancer Center of Excellence, Gynecologic Surgery and Obstetrics, Uniformed Services University and Walter Reed National Military Medical Center, 8901 Wisconsin Avenue, Bethesda, MD 20889 USA; 4 Department of Pathology, Inova Fairfax Hospital, 3300 Gallows Road, Falls Church, VA 22042 USA; 5 Department of Biostatistics, Bioinformatics, and Biomathematics, Lombardi Comprehensive Cancer Center, Georgetown University Medical Center, Washington, DC USA; 6 Theralink Technologies, Inc., 15000 W 6th Ave, Golden, CO 80401 USA; 7 The Henry M. Jackson Foundation for the Advancement of Military Medicine, Inc., 6720A Rockledge Drive, Suite 100, Bethesda, MD 20817 USA; 8 Center for Applied Proteomics and Molecular Medicine, George Mason University, Manassas, VA 20110 USA

**Keywords:** Pancreatic adenocarcinoma, HER2, Proteomics, Laser microdissection

## Abstract

Metastatic pancreatic adenocarcinoma (PDAC) is the third leading cause of cancer-related death in the United States, with a 5-year survival rate of only 11%, necessitating identification of novel treatment paradigms. Tumor tissue specimens from patients with PDAC, breast cancer, and other solid tumor malignancies were collected and tumor cells were enriched using laser microdissection (LMD). Reverse phase protein array (RPPA) analysis was performed on enriched tumor cell lysates to quantify a 32-protein/phosphoprotein biomarker panel comprising known anticancer drug targets and/or cancer-related total and phosphorylated proteins, including HER2^Total^, HER2^Y1248^, and HER3^Y1289^. RPPA analysis revealed significant levels of HER2^Total^ in PDAC patients at abundances comparable to HER2-positive (IHC 3+) and HER2-low (IHC 1+ /2+ , FISH−) breast cancer tissues, for which HER2 screening is routinely performed. These data support a critical unmet need for routine clinical evaluation of HER2 expression in PDAC patients and examination of the utility of HER2-directed antibody–drug conjugates in these patients.

**Supplementary Information:**

The online version contains supplementary material available at 10.1186/s12014-024-09476-7.

## Introduction

Metastatic pancreatic adenocarcinoma (PDAC) is the third leading cause of cancer-related death in the United States [1], with a 5-year survival rate of only 11% [2]. Standard first-line treatment options include gemcitabine plus albumin-bound paclitaxel (gemcitabine/nab-paclitaxel), and 5-fluorouracil (FU)/leucovorin, irinotecan, oxaliplatin (FOLFIRINOX) [3]. Second-line treatment with 5-FU plus liposomal irinotecan has a response rate of less than 20% with median overall survival of 6.2 months [4]. An urgent unmet need exists for identification of novel effective treatment paradigms.

Historically, HER2-directed therapy was only indicated for HER2-positive breast cancer patients, defined as those with HER2 immunohistochemistry (IHC) 3+ , or IHC 2+ and positive fluorescence in situ hybridization (FISH; HER2/CEP17 ratio ≥ 2.0 and *HER2* copy number (CN) signals/cell ≥ 4). Recent studies have shown the analytical and technical limitations of IHC-based quantitation of HER2 protein expression in *HER2* unamplified/negative tumors due to the limitations and variability of interpretation by pathologists and the lack of accuracy and precision of HER2 protein quantitation using current HER2 IHC-based assays in the *HER2* unamplified setting [5–7]. Moreover, recent data has found that approximately 60% of patients who were previously classified as HER2-negative could be considered as HER2-low, defined as IHC 1+ /2+ and FISH-negative [8, 9]. The DESTINY-Breast04 trial (NCT03734029) evaluation of trastuzumab deruxtecan (T-DXd) in previously-treated HER2-low metastatic breast cancer patients revealed that treatment with HER2-directed therapy was associated with significantly longer modified progression-free survival (mPFS) and overall survival (mOS) compared to physician’s choice chemotherapy (9.9 months vs 5.1 months, and 23.4 months vs 16.8 months, respectively) [10]. Data from HER2 IHC 0 metastatic breast cancer patients in the DAISY trial (NCT04132960) also assessing T-DXd response revealed a mPFS of 4.2 months in this population, compared to 6.7 months in HER2-low and 11.1 months in HER2 3+ patients [11]. The DESTINY-Breast04 and DAISY trials have raised interest in predicting who among HER2-low patients are responders to HER2-directed therapy. Aberrant activation (phosphorylation) of the human epidermal growth factor 2 (HER2) receptor tyrosine kinase leads to tumor cell proliferation, migration, and survival [12]. Phosphorylated HER2 is predictive of response to HER2-directed therapeutics in breast cancer [13–16], and specifically in the I-SPY2 TRIAL to HER2-directed tyrosine kinase inhibitors (TKI; such as neratinib) in HER2-negative/unamplified tumors [17], and to antibody drug conjugates (ADCs; such as trastuzumab emtansine) in HER2-positive/amplified tumors [18].

The tumor microenvironment (TME) represents a complex milieu of heterogeneous cell types, including (but not limited to) tumor cells, stromal cells, immune cells, endothelial cells, and a complex extracellular matrix [19]. Several recent studies have demonstrated widespread misinterpretation [20] of gene expression signatures from heterogenous bulk tissue specimens, as several of the genes or proteins correlative with outcomes are stromally expressed [21–26]. Moreover, recent studies have shown that upfront cellular enrichment of tumor epithelium from tissue biopsies is required for accurate determination of total and phosphoprotein expression in tumor epithelium [27, 28]. Selective harvest of discrete cellular subpopulations from the complex milieu of the tumor microenvironment (TME) via laser microdissection (LMD) prior to reverse phase protein array (RPPA) analysis allows for the quantitative measurement and functional assessment of the activation state of protein drug targets and other known cancer-related proteins directly from enriched populations of microdissected tumor cells [28, 29]. IHC-based determinations of HER2 expression in the HER2-low (IHC 1+ /2+ , FISH-negative) or HER2-ultra low (IHC 0) were not analytically accurate when compared to a CLIA quantitative RPPA-based HER2 assay to determine protein expression [7]. Nearly 40% of estrogen receptor (ER)-positive HER2 IHC 0 breast cancer tumors, and 30% of ER-negative HER2 IHC 0 breast cancer tumors expressed modest to moderate amounts of HER2 by RPPA, which approximates a 30% response rate seen with T-DXd in HER2-low patients in the DAISY trial [11].

While HER2 abundance is not routinely evaluated in PDAC patients, previous small cohort studies have shown that HER2 overexpression is observable in an estimated 11–45% of pancreatic carcinoma specimens [30], though clinical trials evaluating HER2-directed therapy have not demonstrated improved outcomes compared to standard chemotherapy [31]. Further, the discrepancy in the prevalence of HER2-positive/amplified tumors in PDAC [30, 31] may be driven by interobserver variability, necessitating the need for more quantitative and reproducible methodologies for HER2 quantification, such as by RPPA. Given the poor treatment options for pancreatic cancer and the efficacy of HER2-directed therapy in HER2-low breast cancer, the findings of RPPA analysis of LMD enriched tumors could represent a new treatment option in this patient population. Here we report RPPA-based quantitative expression and phosphorylation of HER2/3 in LMD enriched tumor samples from patients with pancreatic cancer, breast cancer, and other solid tumor malignancies.

## Methods

### Patient specimens

Formalin-fixed, paraffin-embedded (FFPE) primary and/or metastatic tumor surgical biopsy specimens were prospectively obtained from 14 patients with PDAC, 14 patients with breast cancer, and 40 patients with other solid tumor malignancies as part of an IRB-approved Molecular Tumor Board study at the Inova Schar Cancer Institute (ISCI). Written informed consent was obtained from all patients. All experimental protocols involving human data in this study were in accordance with the Declaration of Helsinki.

Serial tissue thin sections (8 µm) were sectioned by microtome (median = 2 sections, range 2–8), placed onto polyethylene naphthalate (PEN) membrane slides (Leica Microsystems, Wetzlar, Germany), and stained with hematoxylin and eosin (H&E). One representative section (5 µm) per specimen block was mounted onto charged glass slides and H&E stained. Representative slides (the H&E section on glass and one LMD slide per specimen block) were scanned using the Aperio ScanScope XT slide scanner (Leica Microsystems). Micrographs of the representative sections on glass were reviewed by board-certified pathologist in Aperio eSlide Manager and Aperio ImageScope (Leica Microsystems) to annotate and confirm tumor areas of tumor epithelium for LMD enrichment.

### Laser microdissection

LMD enrichment of tumor epithelium (5–10 µm^2^) was performed on the LMD7 (Leica Microsystems). The LMD enriched tumor samples for RPPA analysis were collected into cylindrical pressure cycling technology (PCT; Pressure Biosciences, Inc., South Easton, MA, USA) microtubes. A lysis/extraction buffer containing final concentrations of 10% tris(2-carboxyethyl)phosphine (TCEP, 50 mM; ThermoFisher Scientific, Inc., Waltham, MA, USA), 22.5% tris hydrochloride (Tris–HCl, 225 mM; ThermoFisher Scientific, Inc.), 4% sodium dodecyl sulfate (SDS, *v/v*; ThermoFisher Scientific, Inc.), 10% glycerol (*v/v*; ThermoFisher Scientific, Inc.), and 37.5% MilliQ type I water was added at a ratio of 2.5 µl buffer per mm^2^ LMD tissue. The microtubes were capped with PCT microcaps and placed into 0.5 ml PCR tubes, briefly centrifuged, and stored at − 80℃ until sample lysis. Representative images before and after LMD were captured using the Aperio AT2 slide scanner (Leica Microsystems).

### CLIA-based reverse phase protein microarray analysis

The LMD enriched tumor samples were heated at 95 ℃ for 20 min, briefly centrifuged, and heated at 80 ℃ for 2 h. After heating, the samples were stored at 4 ℃ for one minute and then centrifuged for 18,000 rcf for 15 min. The lysate supernatants containing the denatured proteins were transferred to fresh low protein binding o-ring screw-top tubes (Agilent Technologies, Savage, MD, USA) and stored at − 80 ℃ until shipment on dry ice to Theralink Technologies, Inc. (Golden, CO, USA) for RPPA analysis.

RPPA analysis was performed as previously described [32] using a 32-marker, CLIA certified and CAP accredited commercial calibrated assay panel for examination of the total and phosphoprotein abundances of several targets with known relevance in solid tumors [29, 33]. The CLIA and CAP accredited RPPA assay is a calibrated immunoassay format that has been previously described [34–36], and uses a series of calibrators and controls along with a reference population data set of levels of expression of each of the 32 analytes including total and phosphorylated HER2, phosphorylated HER3, etc. derived from LMD enriched tumor epithelium over 400 FFPE breast cancer tumor biopsy samples (100 of each HER2/HR subtype). The HER2 expression status of the 400 tumors was derived from central lab based HER2 and HR IHC and/or FISH analysis and each experimental sample is interpolated/extrapolated to the reference population using the calibrator sets which are printed on every slide. Under CAP guidelines and in keeping with antibody validation criteria outlined by the RPPA society and now widely used by the RPPA community [37], the RPPA assay workflow includes extensive validation of all antibodies by western blotting, peptide competition and using positive and negative lysates derived from ligand stimulated cell lines. Of note, the CLIA IHC HER2 assay (NeoGenomics, Laboratories, Inc., Fort Myers, FL, USA) uses the Ventana 4B5 clone, which is validated for use as the breast and gastric companion diagnostic. The CLIA RPPA HER2 assay (Theralink Technologies, Inc.) uses the Invitrogen SP3 clone (rabbit recombinant antibody, (catalog MA5-14509, dilution 1:400). However, while the RPPA and IHC assays use different clones, the reference population used by the RPPA CLIA laboratory to qualify the RPPA assay results were derived from 400 breast tumors whose HER2 IHC was previously determined using the 4B5 clone and the results were > 95% correlated [7].

Briefly, sample lysates for RPPA analysis were printed onto nitrocellulose backed slides in technical triplicates alongside all requisite controls and calibrators. Prior to staining, nitrocellulose slides were pre-treated with ReBlot (MilliporeSigma, Rockville, MD, USA) and a blocking reagent (I-Block; Applied Biosystems, Waltham, MA, USA). Slides were incubated with primary antibodies for 30 min at room temperature, followed by incubation with a secondary antibody. Each staining run included a single negative control slide (only antibody diluent; no primary antibody). Protein detection was amplified via horseradish peroxidase mediated biotinyl tyramide deposition and visualized using a fluorescent probe (LI-COR Biosciences, Inc., Lincoln, NE, USA). Images of the stained RPPA slides were captured on an InnoScan 710-AL (Innopsys, Inc., Chicago, IL, USA). Individual spots were detected using the InnoScan software program, Mapix, and spots with non-standard morphology and/or staining were flagged and removed from analysis. The average median intensity value was calculated for each sample on the array for each analyte as well as the negative control slide. Background subtracted intensity values for each sample were fit to an analyte-specific calibrator and total protein normalized. Resulting values were compared to a population reference to determine patient sample percentile and quartile score. The RPPA data is provided in the report as both a continuous variable percentile-based output (0–100) as well as a quartile-based output (0–3) based on the relationship of the patient-specific value compared to the population reference. The 0–3 scoring output is meant to provide interpretive context to physicians who are most used to looking at CLIA IHC-based scoring outputs for protein biomarkers used in patient treatment decision making (e.g. PD-L1, HER2, etc.).

### Next generation sequencing

Clinical NGS (specifically, DNA-seq) analysis was performed using the Tempus xT 648-gene panel [38] (Tempus Labs, Inc., Chicago, IL, USA), prioritizing the same FFPE tissue specimen as was used for the LMD-RPPA analysis whenever possible. Matched specimens (ie. the same specimen block) were available for 46 patients for analysis by both RPPA and DNA-seq. Different specimen blocks were used for RPPA versus DNA-seq for 20 patients. Different specimen blocks were used for RPPA versus DNA-seq for 20 patients. The samples for DNA-seq were prepared from tissue sections with a median of 50% tumor cellularity after macrodissection/microdissection (range = 20–90% tumor cellularity).

### Bioinformatic and statistical analysis

Descriptive statistics was used to summarize the patients' demographics. Fisher's Exact tests were used to compare the RPPA abundances of HER2^Total^, pHER2^Y1248^, and pHER3^Y1289^ between groups. SAS software (v9.4; SAS Institute, Cary, NC, USA) was used for statistical analysis. A significance threshold of p < 0.05 was considered statistically significant. Boxplots of RPPA normalized intensity scores for HER2^Total^, pHER2^Y1248^, and pHER3^Y1289^ were generated using BoxPlotR [39].

## Results

**Fig. 1:**
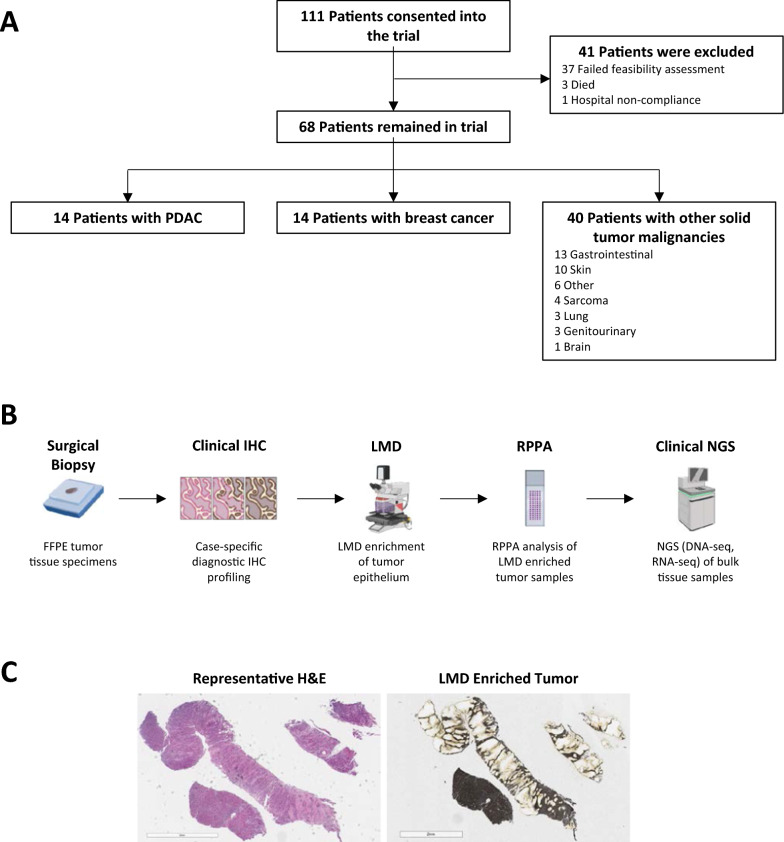
Overview of study design. **A** Formalin-fixed, paraffin-embedded (FFPE) primary and/or metastatic tumor surgical biopsy specimens were prospectively obtained from patients with PDAC, breast cancer, and other solid tumor malignancies as part of an IRB-approved Molecular Tumor Board (MTB) study at the Inova Schar Cancer Institute (ISCI). **B** Workflow diagram depicting tissue specimen analysis. Tumor epithelium (5–10 µm^2^) from tissue specimens was harvested via LMD at a ratio of 1 mm^2^ LMD area per 2.5 µl buffer into a lysis buffer containing 50 mM Bond-Breaker TCEP Solution, 225 mM Tris–HCl, 4% *v/v* sodium dodecyl sulfate (SDS), 10% *v/v* glycerol, in MilliQ Type I water. RPPA analysis of the LMD enriched tissue lysates was performed by Theralink Technologies, Inc., as previously described [32, 33]. **C** Representative micrographs of tissue thin sections stained with hematoxylin and eosin (H&E) mounted onto a glass slide histopathological assessment (left, 5 µm), and onto a slide containing a polyethylene naphthalate (PEN) membrane after LMD harvest of tumor epithelium (right, 8 µm). Clinical NGS was performed by a commercial sequencing laboratory

From October 13, 2021 until December 8, 2022, a total of 111 patients were consented to an IRB-approved study examining the feasibility and utility of including quantitative proteomic analysis into a Molecular Tumor Board (MTB) setting at the Inova Schar Cancer Institute (ISCI) (Fig. 1A). The cohort analyzed in the present subsidiary analysis was comprised of primary and/or metastatic tissue specimens from a cohort of 14 patients with PDAC, 14 patients with breast cancer, and 40 patients with other solid tumor malignancies were selected (Additional file 1: Table S1).

Tumor epithelium was harvested via LMD for RPPA quantification of total HER2 (HER2^Total^), phosphorylated (p)HER2^Y1248^, and pHER3^Y1289^ as part of a 32-protein/phosphoprotein biomarker, research use only (RUO) panel in a commercial CLIA/CAP-accredited laboratory (Theralink Technologies, Inc.) examining the abundances of targets with known relevance in solid tumors, as previously described (Fig. 1B, C) [7, 16–18, 28, 29, 32, 33]. Protein and phosphoprotein-level expression was determined by comparing the RPPA-derived quantitative values from each patient LMD tumor sample in our cohort against an existing and extensively validated RPPA reference dataset derived from LMD enriched tumor epithelium from 400 breast cancer specimens with centrally-determined HER2-positive/amplified (IHC 3+ /2+ , FISH-positive) and HER2-negative/unamplified (IHC 0/1+ /2+ , FISH-negative) status. In keeping with the RPPA as a clinical immunoassay format, the known HER2^Total^, pHER2^Y1248^, and pHER3^Y1289^ expression in the validated reference population was used to calculate the proteomic/phosphoproteomic expression of these targets from the LMD enriched samples from our cohort of patients with pancreatic, breast, or other solid tumors using the external calibrators printed on every slide. The RPPA HER2^Total^, pHER2^Y1248^, and pHER3^Y1289^ continuous variable percentile values (0–100), the RPPA 0–3 intensity score and the HER2 IHC value (when obtained), are provided for each of the tumor samples from the 68 patients, including the HER2-positive and HER2-negative breast cancer tumors so a detailed cohort level and case-by-case comparison between the RPPA percentile and categorical HER2^Total^ values, the pHER2^Y1248^ and pHER3^Y1289^ values, and the IHC determined HER2 values can be seen (Additional file 1: Table S2). Clinical next generation sequencing (NGS; DNA-seq and RNA-seq) of bulk tissue specimens was performed by a commercial sequencing laboratory, prioritizing the same specimen block that was used for LMD-RPPA when possible.

**Fig. 2:**
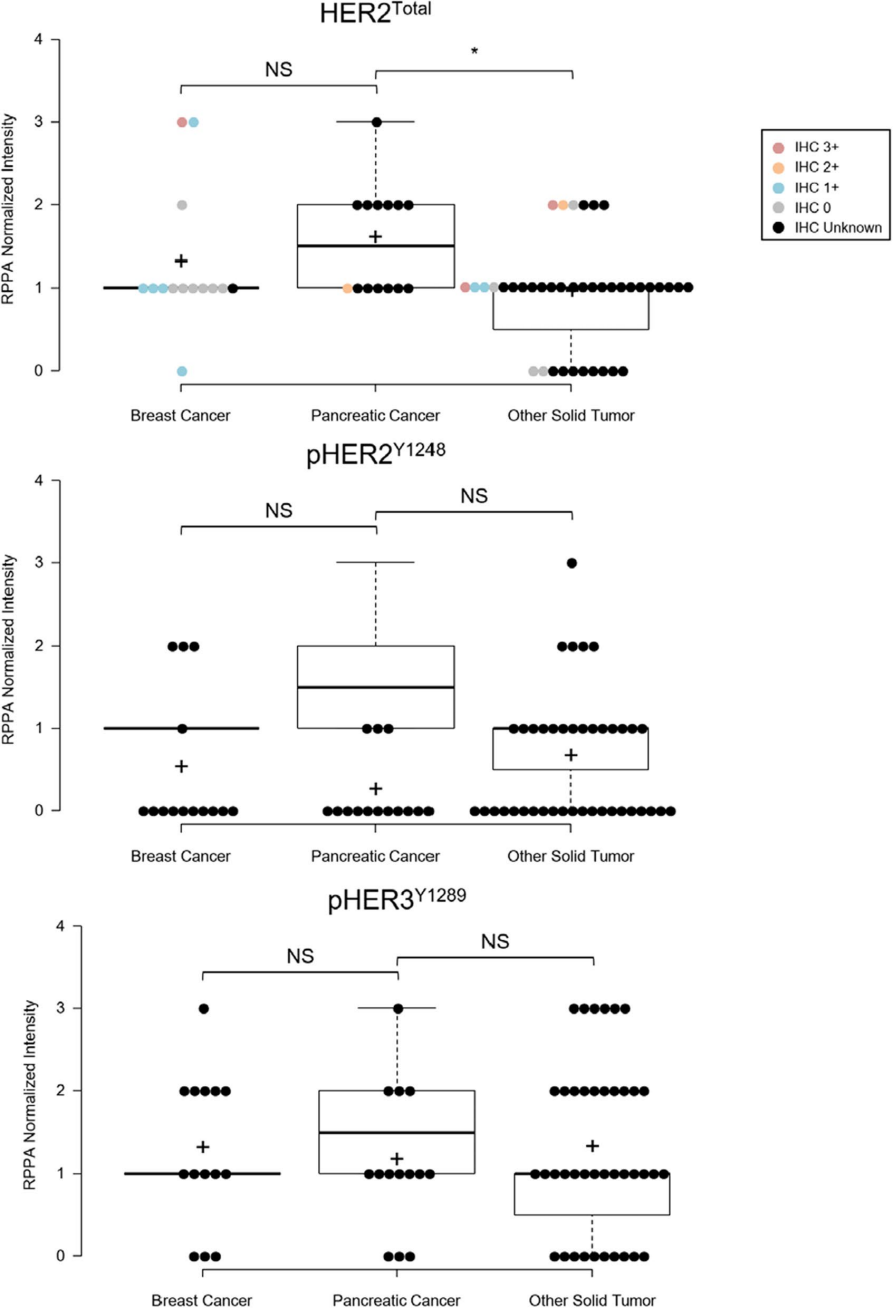
RPPA quantification of HER2^Total^, pHER2^Y1248^, pHER3^Y1289^ and correlation with CLIA-approved clinical IHC scoring. Fisher's Exact tests were performed using SAS software (v9.4) to compare the RPPA abundances of HER2^Total^, pHER2^Y1248^, and pHER3^Y1289^ between groups. An asterisk (*) indicates p < 0.05. *NS*  not significant

PDAC tumors had significantly higher relative levels of HER2^Total^ expression than other solid tumors (p = 0.0112), and trended higher than the predominantly HER2-negative breast cancers in our cohort (p = 0.0962) (Fig. 2, Additional file 1: Table S3). Moreover, since a heterogeneous HER2-positive and HER2-negative breast cancer population was used as a reference, the PDAC HER2^Total^ levels in this cohort are comparable to those from breast cancer patients with HER2-positive and HER2-low tumors. The mean HER2^Total^ abundances in PDAC, breast, and all other solid tumor malignancies were 1.6, 1.3, and 0.9, respectively (Fig. 2). Activated pHER2^Y1248^ and pHER3^Y1289^ abundances did not differ between patients with PDAC vs all other solid tumors or between patients with PDAC vs breast cancer (p > 0.05). RNA-seq revealed *ERBB2* (*HER2*) overexpression in four PDAC patients, one breast cancer patient, and two patients with older solid tumors. *ERBB2* CN gain by DNA-seq was not observed in the patients with PDAC or breast cancer, consistent with previous studies [30]. *ERBB2* CN gain was measured in three other solid tumor patients (gastric and esophageal). Activating *ERBB2* mutations were measured in specimens from two breast cancer patients (S310F, G776V) and one other solid tumor patient (R929W).

HER2 IHC analysis was independently performed for 23 patients using the same specimens as were used for the LMD-RPPA analysis (Additional file 1: Table S3). The HER2^Total^ abundances quantified by IHC and RPPA were not correlated, consistent with previous findings [7]. Further, RPPA-based quantification of HER2^Total^ and pHER2^Y1248^ were not correlated in PDAC or breast cancer tumors.

## Discussion

IHC evaluation of HER2 expression is not a routine part of clinical care for PDAC. Based on conventional criteria of 3 + IHC staining or 2 + IHC with FISH-positivity, less than 2% of PDAC are classified as HER2-positive [40]. Routine NGS profiling for PDAC patients is performed to evaluate *ERBB2* amplification, which is found in only 2% of cases [41]. Since systemic therapies for PDAC have poor results and newer HER2-targeting agents, including ADCs, are benefitting patients HER2-low tumors, there is a critical unmet need to identify PDAC patients who may benefit from anti-HER2 therapy [10]. A few prior studies have demonstrated efficacy of HER2-directed therapy in HER2-overexpressed PDAC cell lines [42] and single-patient reports [43].

We demonstrate a high rate of HER2^Total^ expression in PDAC tumors, with more than half of the tumors showing modest to moderate levels of RPPA-determined HER2^Total^. Moreover, RPPA-determined HER2^Total^ expression in our PDAC cohort was higher than in other solid tumors, and most significantly, in over half of the population studied. Importantly, the HER2^Total^ protein levels measured quantitatively by RPPA in PDAC are comparable with HER2^Total^ abundances in HER2 IHC 3 + amplified breast cancer, as well as HER2-low breast tumors. RPPA-determined tumor HER2 and pHER2 correlations with HER2-therapy based clinical treatment response have not yet been described in PDAC, so we sought to utilize what we postulated was the most conservative approach to defining the context of HER2 and pHER2 expression and activation in PDAC, which was contextualizing the RPPA based HER2 and pHER2 levels to a breast cancer reference comprised of known CLIA IHC/FISH determined *HER2* unamplified (including HER2-low tumors) and *HER2* amplified tumors. Since the CLIA RPPA is a calibrated assay and since both the breast tumor reference and PDAC patient input were both derived from LMD enriched tumor epithelium that were arrayed at the same protein concentrations, a direct comparison can be made, allowing us to determine and compare the relative levels of HER2 protein expression and HER2 activation (phosphorylation) in PDAC compared to *HER2* unamplified breast cancers. This is critical since these breast cancer patients with HER2-low disease are now being routinely treated under FDA approved therapeutic regimens with anti-HER2 agents, which allows us to contextualize the major findings of our paper that over 50% of PDAC patients have tumors that express the same levels of HER2 protein and HER2 protein activation as the tumors from breast cancer patients who are now routinely prescribed HER2 therapies on-label because of those HER2 levels.

These data suggest that while the inhibition of HER2 signaling by a TKI such as lapatinib or tucatinib will be unlikely to provide clinical benefit as the HER2 pathway does not appear to be highly activated in PDAC patients, the use of HER2-targeting ADCs such as T-DXd may promote directed delivery of drugs to the specific HER2-expressing tumor cells. Concordantly, interim analysis from DESTINY-PanTumor02 phase II trial reported meaningful responses to T-DXd in patients with PDAC [44]. Our results further suggest that future investigations of HER2-targeting ADCs, including T-DXd, in HER2-low PDAC patients will have a large pool of potential patients eligible for enrollment. The HER3 abundance and activation state demonstrated here may also provide another avenue for exploration, though HER3 has not been established as a predictive biomarker [45].

A streamlined LMD-RPPA analytical workflow uniquely allows for direct quantification of HER2^Total^ abundance and activation state. This same workflow has been found to identify HER2 expressing breast cancers in the HER2-low setting missed by IHC [18] and could be a new molecular approach to identify PDAC patients whose tumor HER2 levels are druggable by new ADC therapeutics. The HER2-positive arm for biliary tract malignancies in TAPUR demonstrated responses in patients with activating *HER2* mutations, in addition to amplified or overexpressed patients [46]. Phosphoprotein activation analysis may be useful for predicting response in this subset, in agreement with prior studies demonstrating that HER2 phosphorylation could predict response to neratinib in HER2-mutated breast and NSCLC patients [47]. Given the redundancies and complexity of signaling pathways, in a pan-tumor setting it is likely that some potential responders will not have overexpression, amplification, or activating mutations, thus both quantitative HER2^Total^ and pHER2 functional protein signaling pathway mapping via RPPA remains a compelling tool for predicting responsiveness to HER2-targeted ADC and/or TKI therapeutics. Additionally, LMD enrichment of specimens allows for tumor-centric proteomic and phosphoproteomic profiling for a more accurate assessment of HER2 abundance, which may explain the discrepancy between the HER2^Total^ by RPPA and the HER2 IHC characterization determined through conventional laboratory diagnostics.

The primary limitation of our study is the number of patients. Additionally, the clinical relevance of HER2 phosphorylation and quantitative HER2^Total^ measurements in PDAC remains unknown, as no prospective studies to date have utilized RPPA analysis in PDAC patients to examine HER2^Total^ or pHER2/3 as predictive biomarkers. Lastly, LMD is not widely available in the clinical setting. Nonetheless, we were encouraged by the high percentage of PDAC tumors in which HER2 was expressed and/or activated. Further studies quantifying proteomic HER2 expression and/or activation in larger patient cohorts are ongoing. Given the dearth of options for relapsed/refractory PDAC, this is a fertile ground for exploration.

In conclusion, we demonstrate that in over half of the PDAC patient tumors evaluated in our study, HER2^Total^ protein expression levels are comparable to that of breast cancer patients, for which HER2 expression is routinely evaluated and now therapeutically targeted with FDA-approved HER2-directed agents. While our evaluation of phosphoprotein expression demonstrated overall low activation rates (pHER2^Y1248^ and pHER3^Y1289^), some patients with activated HER2 phenotypes were also found who could be sensitive to HER2-directed TKI therapy as well. More studies are needed to evaluate the clinical benefit of targeting HER2 in this population.

### Supplementary Information

**Additional file 1: Table S1.** Demographic and clinical characteristics of the patients. **Table S2.** The HER2^Total^, pHER2^Y1248^, and pHER3^Y1289^ abundances quantified by RPPA are reported as categorical values of 0, 1, 2, and 3, as well as normalized percentile values ranging from 0-100% relative to the reference population standards. The IHC-based characterizations of HER2^Total^ are reported as quartile-based categorical values of 0, 1, 2, and 3, per standard clinical scoring, for which an IHC score of 3+ or 2+/FISH-positive is clinically defined as HER2-positive. Clinical IHC-based HER2^Total^ testing was not performed for all tumors; those tumors without IHC data are reported as “Not evaluated”. **Table S3.** Spearman correlations were calculated between the HER2^Total^ and pHER2^Y1248^ abundances quantified by RPPA each for PDAC and breast cancer tumors, and between the HER2Total abundances quantified by RPPA and standard clinical IHC staining each for PDAC and breast cancer tumors. ND = not done (IHC staining not performed).

## Data Availability

The datasets used during the current study may be available from the corresponding author on reasonable request providing anonymization can be maintained.

## Abbreviations

PDAC: Pancreatic adenocarcinoma
RPPA: Reverse phase protein array
LMD: Laser microdissection
IHC: Immunohistochemistry
FISH: Fluorescence in situ hybridization
FU: Fluorouracil
CN: Copy number
T-DXd: Trastuzumab deruxtecan
mPFS: Modified progression-free survival
mOS: Overall survival
HER2-: Human epidermal growth factor 2
TKI: Tyrosine kinase inhibitors
ADCs: Antibody drug conjugates
TME: Tumor microenvironment
ER: Estrogen receptor
MTB: Molecular Tumor Board
ISCI: Inova Schar Cancer Institute
p: Phosphorylated
